# Artificial intelligence-based graded training of pulmonary nodules for junior radiology residents and medical imaging students

**DOI:** 10.1186/s12909-024-05723-5

**Published:** 2024-07-09

**Authors:** Xiaohong Lyu, Liang Dong, Zhongkai Fan, Yu Sun, Xianglin Zhang, Ning Liu, Dongdong Wang

**Affiliations:** 1 Department of Radiology, The First Affiliated Hospital of Jinzhou Medical University, Jinzhou, China; 2 School of Electrical Engineering, Liaoning University of Technology, Jinzhou, China; 3 Office of Educational Administration, The First Affiliated Hospital of Jinzhou Medical University, Jinzhou, China

**Keywords:** Artificial intelligence, Pulmonary nodule, Medical imaging, Training

## Abstract

**Background:**

To evaluate the efficiency of artificial intelligence (AI)-assisted diagnosis system in the pulmonary nodule detection and diagnosis training of junior radiology residents and medical imaging students.

**Methods:**

The participants were divided into three groups. Medical imaging students of Grade 2020 in the Jinzhou Medical University were randomly divided into Groups 1 and 2; Group 3 comprised junior radiology residents. Group 1 used the traditional case-based teaching mode; Groups 2 and 3 used the ‘AI intelligent assisted diagnosis system’ teaching mode. All participants performed localisation, grading and qualitative diagnosed of 1,057 lung nodules in 420 cases for seven rounds of testing after training. The sensitivity and number of false positive nodules in different densities (solid, pure ground glass, mixed ground glass and calcification), sizes (less than 5 mm, 5–10 mm and over 10 mm) and positions (subpleural, peripheral and central) of the pulmonary nodules in the three groups were detected. The pathological results and diagnostic opinions of radiologists formed the criteria. The detection rate, diagnostic compliance rate, false positive number/case, and kappa scores of the three groups were compared.

**Results:**

There was no statistical difference in baseline test scores between Groups 1 and 2, and there were statistical differences with Group 3 (*P* = 0.036 and 0.011). The detection rate of solid, pure ground glass and calcified nodules; small-, medium-, and large-diameter nodules; and peripheral nodules were significantly different among the three groups (*P*<0.05). After seven rounds of training, the diagnostic compliance rate increased in all three groups, with the largest increase in Group 2. The average kappa score increased from 0.508 to 0.704. The average kappa score for Rounds 1–4 and 5–7 were 0.595 and 0.714, respectively. The average kappa scores of Groups 1,2 and 3 increased from 0.478 to 0.658, 0.417 to 0.757, and 0.638 to 0.791, respectively.

**Conclusion:**

The AI assisted diagnosis system is a valuable tool for training junior radiology residents and medical imaging students to perform pulmonary nodules detection and diagnosis.

## Background

With the development of imaging technology and the improvement of health awareness among pepole, the detection rate of pulmonary nodules has been steadily increasing. A few studies have indicated that pulmonary malignant tumors, tuberculosis, metastatic tumors, hamartoma, inflammatory pseudotumors, sarcoidosis, etc. these etiologies can present with pulmonary nodules [1, 2]. According to the 2020 data of the International Agency for Research on Cancer [3], the incidence of lung cancer ranks the second among malignant tumors, and the mortality ranks the first. Early lung cancer often presents as solitary pulmonary nodules. The prognosis of pulmonary nodules of different natures is extremely different. In 2021, the World Health Organization’s lung tumors guidelines [4] defined that carcinoma in situ and atypical adenomatoid hyperplasia as precursor gland lesions. Accurate diagnosis and corresponding treatment at the early stage of lung cancer is crucial for disease control, prognosis, and improving the survival rate of patients [5]. Currently, there is no non-invasive method to clinically differentiate benign and malignant pulmonary nodules. Thus, ascertaining the nature of pulmonary nodules remains a complicated issue.

Recently, the wide application of artificial intelligence (AI) in various medical fields has promoted the rapid development of precision medicine [6]. AI assisted diagnosis systems have a favourable impact on the detection of pulmonary nodules, which can significantly reduce the rate of missed diagnosis of pulmonary nodules [7, 8]. Many studies have explored the application of AI technology algorithm to diagnose pulmonary nodules. This technology could help radiologists rapidly and accurately detect pulmonary nodules, which could be beneficial for pulmonary nodule screening [9]. However, few studies have examined its effect on the clinical practice of junior radiology residents and medical imaging students. This study explores whether AI-based software training could help improve the detection efficiency and clinical application value of pulmonary nodules for junior radiology residents and medical imaging students. This would help cultivate medical imaging professionals who could adapt to the requirements of intelligent social technology and medical development.

## Methods

### Group design

Participants were randomly recruited from amongst interested first-year radiology residents and medical imaging students entering clinical studies at the Jinzhou Medical University. Based on different methods of picture training, they were divided into three groups. Group 1: six five-year medical imaging students of Grade 2020 who read pictures independently with the picture archiving and communication system (PACS). Group 2: six five-year medical imaging students of Grade 2020 who read pictures with AI. Group 3: six junior radiology residents who read pictures with AI. Groups 1 and 2 had recently completed the theoretical learning of lung imaging diagnosis before enrollment. Before the training, all participants in the three groups participated in the test related to lung nodule imaging performance. The scores in Groups 1, 2 and 3 were (77.50 ± 8.17), (80.33 ± 7.92) and (89.33 ± 4.50), respectively. There was no statistical difference between Groups 1 and 2(*P* > 0.05). However, the difference between the first two groups and the third group was statistically significant (*P* = 0.036 and 0.011).

### Traing and test

In the non-AI training mode (Group 1), teachers summarised the diagnosis points and cases of pulmonary nodules in 10–15 min. Next, cases pushed to the PACS, where students read images in groups and participated in discussion and diagnosis. Finally, they rectified errors based on comparison with standard case reports.

In the AI-training mode (Groups 2 and 3), teachers summarised the diagnosis points and cases of pulmonary nodules in 10–15 min; thereafter, the students used AI for practical training. First, the operators read and diagnosed the cases independently using the PACS. Next, the AI software was used to acquire and summarise the detailed information marked on the software, such as nodules and lesions, specific positions of lung lobes and lung segments, and the judgment of benign and malignant tumors. Finally, group discussions were conducted to address any misunderstandings or doubts, after which, the teachers addressed students’ queries.

**Fig. 1:**
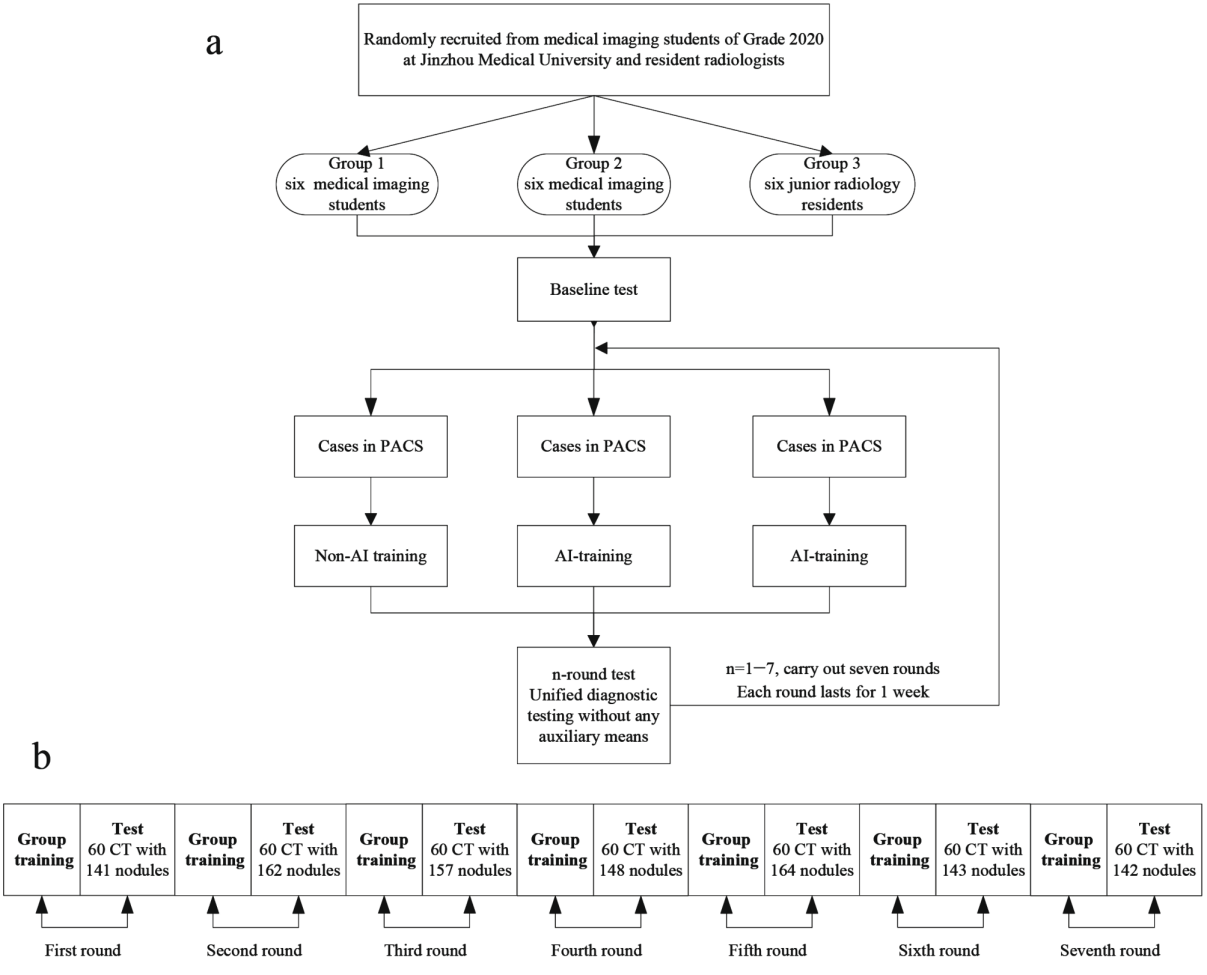
Research flowchart

The duration of each round was one week (two class hour/day); that of the entire process was seven weeks. Figure 1a illustrates the research flowchart.

A total of 420 plain computed tomography (CT) images with 1,057 pulmonary nodules were included in the test, which was performed for seven rounds with 60 images per round. Each round contains benign and malignant nodules. All three groups underwent tests without any auxiliary conditions (Fig. 1b). Inclusion criteria: (1) 1 ≤ number of pulmonary nodules per case ≤ 5; (2) Nodules with diameter between 3 and 30 mm [10]; and (3) Thickness of CT-reconstructed image 1 mm. Exclusion criteria: (1) Diffuse lung lesions; (2) Previous pulmonary surgery history; (3) Multiple lung metastases; and (4) Artifacts or falling effects in the images.

### Observations and grading methods

The diameter, shape, edge, density, boundary, vacuole or cavity, lobulation sign, pleural depression sign, vascular cluster sign and other signs of pulmonary nodules were observed. Based on the signs, benign and malignant diagnosis was made. Diagnosis of pulmonary nodules was graded according to the Lung CT Screening Reporting and Data System [11, 12] (Lung-RADS) (Table 1).

**Table 1:** The grading standard of Lung-RADS

Category	Grading standard	
1	No nodules or definitely benign nodules	
2	Nodules with a very low suspicion of developing lung cancer and a likelihood to be malignant < 1%	
3	Nodules with some suspicion of being benign but with a likelihood to be malignant ≥ 1%, requiring follow-up observation	
4 A	Nodules suspected to be lung cancer with a likelihood to be malignant of 5–15%, requiring additional testing or biopsy	
4B	Nodules suspected to be lung cancer with a likelihood to be malignant > 15%, requiring further testing or biopsy	
4 C	Nodules suspected to be lung cancer with a high probability, the lesions persisted with some solid components not improving, or growing and burring	
5	Nodules suspected to be lung cancer strongly, requiring biopsy	
6	Nodules are histologically malignant	

### Classification and criteria

Based on the density of pulmonary nodules [13], they were classified into solid nodule (a nodule that completely covers the lung parenchyma), purely ground glass nodule (pGGN) (a hazy opacity without blocking underlying pulmonary vessels or bronchial structure, no solid components), mixed ground glass nodule (mGGN) (a nodule with ground glass components as well as solid components) and calcified nodule (a nodule with calcium deposition). Based on the location of pulmonary nodules, they were classified into subpleural nodules (connected to the pleura), central nodules (within 20 mm of the hilar), and peripheral nodules (outside the hilar region, but not connected to the pleura). Based on the maximum diameter of nodules, they were divided into small nodules (less than 5 mm), medium nodules (5 to 10 mm), and large nodules (over 10 mm).

Two associate chief physicians with 10 years of experience in chest imaging observed, identified and marked the lesions by referring to AI results on the thin section CT images (1 mm), judged the types of nodules, recorded the size of nodules, and thereafter, ascertained the authenticity of nodules using the multi-planar reconstruction and maximum density projection technology. The consensus of the two doctors and the average size of nodules were taken as the reference standard. In case of different opinions, the senior physician shall be invited to consult for confirmation. For nodules with surgery or puncture, pathological findings were the criteria. For nodules without pathological results, the diagnostic opinions of radiologists were the criteria. Detection rate = (number of true positive nodules/total number of nodules) ×100%. Diagnosis coincidence rate = (number of correctly diagnosed nodules /total number of nodules) ×100%. False positive number/each case = number of false positive nodules/number of cases.

### Introduction to the AI-assisted diagnosis system

**Fig. 2:**
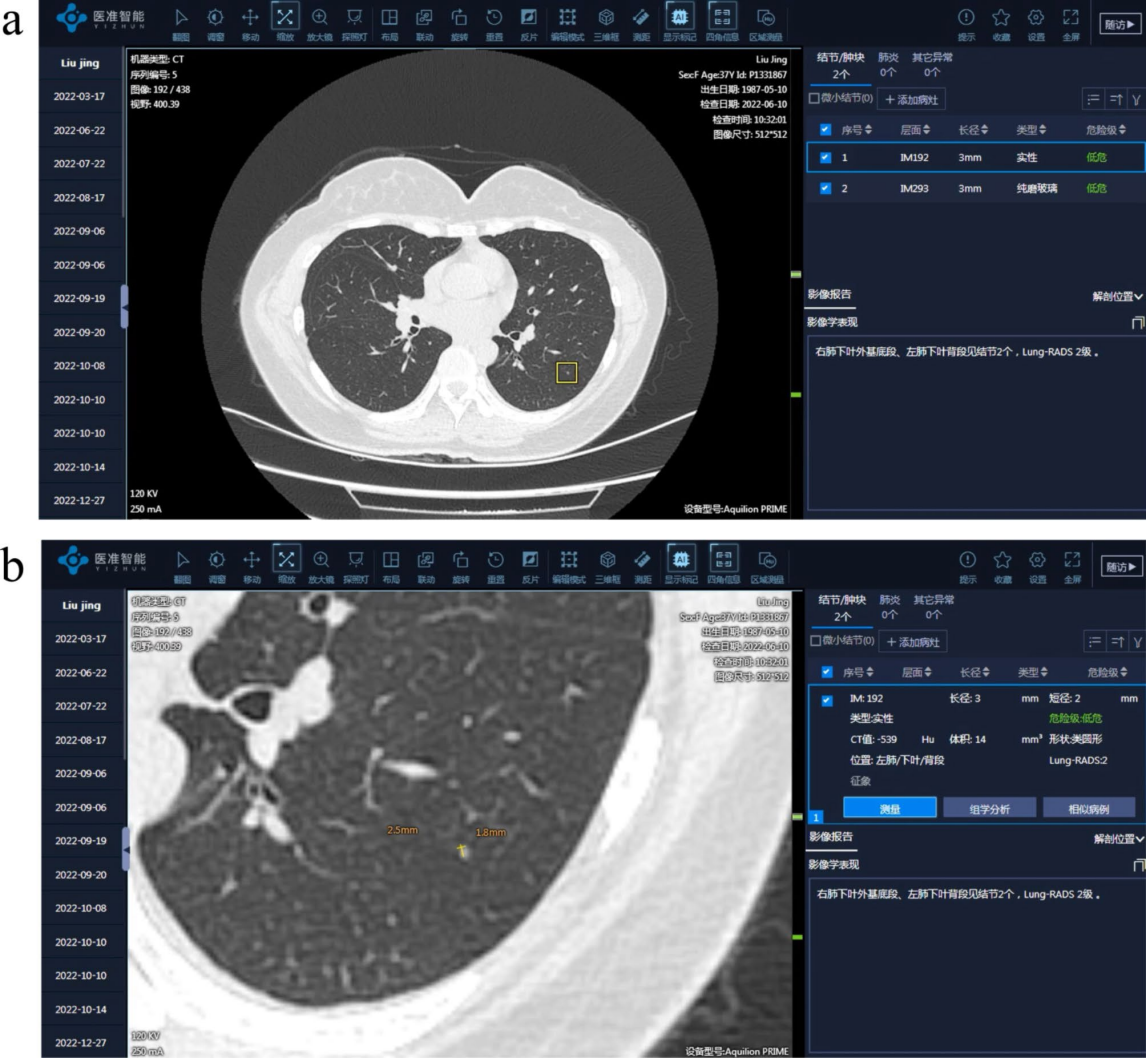
AI assisted diagnosis system interface. (**a**) The marking interface of AI assisted system that can display the location, quantity, type, and grading. information of lung nodules. (**b**) The measurement interface of the system, which further displays the volume and size of nodules based on a

AI film reading was conducted using the medical intelligent image assisted diagnosis software (V4.0 version) of the Beijing Medical Intelligent Technology Co., LTD. The performance of AI alone on test datasets was as follows: sensitivity of 95.3%, specificity of 96.8%. When the complete original CT images are imported into the system, the lung image data can be labelled in batches and could automatically delineate the region of interest to automatically locate, measure and diagnose the pulmonary nodules. In the right column of the system interface, the number of pulmonary nodules, level, diameter, type, location and malignancy of each nodule could be displayed (Fig. 2). Next, a complete AI report can be issued, which could be pushed to the PACS terminal with one click. The system could independently select the longest diameter range of displayed nodules to add or delete nodules, as required. As the inclusion criteria of this study, it is set to detect and diagnose only the nodules with diameter between 3 and 30 mm.

### Statistical analysis

The SPSS 23.0 (IBM, Chicago, USA) software was used for statistical analysis, and GraphPad Prism 8.0 (GraphPad, California, USA) was used for data plotting. Tree diagnosis classifications were set as with/without pulmonary nodule, and benign/malignant nodule. The detection rate, diagnostic compliance rate and false positive number/case of each group were calculated. The counting data were expressed as % and the χ2 test was used for comparison among the groups. Measurements with normal distribution were expressed as mean ± standard deviation ($\mathop {\rm X}\limits^ - \; \pm \;{\rm{s}}$), and the T-test, F-test were performed for inter-group and multi-group comparisons. The Kappa score was used to evaluate the intra-group consistency of the test results of each round for three groups of trainees, which were calculated by combining the average value of the harmonic of the three classifications. The kappa scores between 0.41 and 0.60 were considered general agreement, between 0.61 and 0.80 were considered significant agreement, and above 0.80 were considered highly agreement. The difference was statistically significant with *P* < 0.05.

## Results

### Nodules distribution with different density, size, and location

A total of 1,057 nodules were identified on the CT images of 420 patients, [632 benign nodules (59.8%) and 425 malignant nodules (40.2%)];661 solid nodules (62.5%), 195 pGGN (18.5%) and 53 mGGN (5.0%), 148 calcified nodules (14.0%); 458 small nodules (43.3%), 374 medium nodules (35.4%), 225 large nodules (21.3%); 143 subpleural nodules (13.5%), 852 peripheral nodules (80.6%), and 62 central nodules (5.9%).

### Comparison of the different nodule detection rates, diagnostic compliance rates, and false positive numbers in each group

The detection rates of solid nodules, pGGNs and calcified nodules, small-diameter nodules, medium-diameter nodules, large-diameter nodules, and peripheral nodules were significantly different among the three groups (*P*<0.05). There was no statistically significant difference in the detection rate of mGGNs, subpleural nodules and central nodules among the three groups, as well as those between Groups (*P* > 0.05). The difference between Groups 2 and 3 was statistically significant only in the detection rate and the number of false positive nodules for pGGN. Without AI assistance, the number of false positive nodules in solid nodules, pGGNs, mGGNs, small-diameter nodules, medium-diameter nodules, peripheral nodules, and central nodules in Group 1 was significantly increased (*P*<0.05). There was no statistically significant difference in the number of false positive nodules for calcified, large, and subpleural nodules (*P* > 0.05). Table 2 presents the relevant test values.

**Table 2:** Comparison of pulmonary nodule detection results among the three groups of observers

Group		Solid nodules		pGGN		mGGN		Calcified nodules	
		Detection rate (%)	False positive nodule	Detection rate (%)	False positive nodule	Detection rate (%)	False positive nodule	Detection rate (%)	False positive nodule
1		78.97	0.55 ± 0.27	64.10	0.20 ± 0.07	92.45	0.21 ± 0.10	71.62	0.07 ± 0.04
2		89.71	0.29 ± 0.18	78.46	0.18 ± 0.11	88.68	0.12 ± 0.05	89.86	0.08 ± 0.03
3		91.38	0.26 ± 0.11	82.05	0.32 ± 0.08	96.23	0.08 ± 0.03	89.19	0.05 ± 0.02
entirety	Statistics	51.937^a^	3.900^b^	18.699^a^	4.410^b^	0.541^a^	5.955^b^	23.050^a^	1.448^b^
	*P*	0.000	0.043	0.000	0.031	0.763	0.013	0.000	0.266
1vs2	Statistics	28.874^a^	2.277^c^	9.820^a^	0.392^c^	0.442^a^	2.332^c^	15.840^a^	0.557^c^
	*P*	0.000	0.038	0.002	0.700	0.506	0.034	0.000	0.586
2vs3	Statistics	1.069^a^	0.263^c^	15.965^a^	2.746^c^	1.217^a^	1.037^c^	0.036^a^	1.671^c^
	*P*	0.301	0.796	0.000	0.015	0.270	0.316	0.850	0.115
1vs3	Statistics	40.278^a^	2.539^c^	0.793^a^	2.353^c^	0.177^a^	3.369^c^	14.496^a^	1.114^c^
	*P*	0.000	0.023	0.373	0.033	0.674	0.004	0.000	0.283

Group		Small nodules		Medium nodules		Large nodules	
		Detection rate (%)	False positive nodule	Detection rate (%)	False positive nodule	Detection rate (%)	False positive nodule
1		74.24	0.94 ± 0.42	74.06	0.19 ± 0.10	92.89	0.07 ± 0.02
2		88.86	0.47 ± 0.29	81.02	0.07 ± 0.02	96.44	0.04 ± 0.02
3		90.83	0.42 ± 0.32	84.49	0.09 ± 0.03	95.11	0.05 ± 0.03
entirety	Statistics	67.858^a^	4.082^b^	15.220^a^	6.584^b^	18.793^a^	0.059^b^
	*P*	0.000	0.038	0.000	0.009	0.000	0.943
1vs2	Statistics	38.514^a^	2.341^c^	6.376^a^	3.387^c^	13.912^a^	0.341^c^
	*P*	0.000	0.034	0.012	0.004	0.000	0.738
2vs3	Statistics	0.969^a^	0.249^c^	1.583^a^	0.564^c^	0.495^a^	0.131^c^
	*P*	0.325	0.807	0.208	0.581	0.482	0.898
1vs3	Statistics	50.449^a^	2.590^c^	14.154^a^	2.822^c^	9.688^a^	0.210^c^
	*P*	0.000	0.021	0.000	0.013	0.002	0.837

Group		Subpleural nodules		Peripheral nodules		Central nodules	
		Detection rate (%)	False positive nodule	Detection rate (%)	False positive nodule	Detection rate (%)	False positive nodule
1		74.13	0.10 ± 0.05	76.76	0.39 ± 0.15	67.74	0.49 ± 0.20
2		80.42	0.13 ± 0.02	89.79	0.23 ± 0.11	75.81	0.31 ± 0.15
3		83.22	0.09 ± 0.05	91.31	0.20 ± 0.09	79.03	0.25 ± 0.10
entirety	Statistics	3.771^a^	1.443^b^	90.305^a^	4.398^b^	2.190 ^a^	3.873^b^
	*P*	0.152	0.267	0.000	0.031	0.335	0.044
1vs2	Statistics	1.613^a^	1.225^c^	51.915^a^	2.323^c^	0.995^a^	2.006^c^
	*P*	0.204	0.240	0.000	0.035	0.319	0.063
2vs3	Statistics	0.376^a^	1.633^c^	1.159^a^	0.436^c^	0.185^a^	0.669^c^
	*P*	0.540	0.123	0.282	0.669	0.668	0.514
1vs3	Statistics	3.521^a^	0.408^c^	67.267^a^	2.758^c^	2.023^a^	2.674^c^
	*P*	0.060	0.689	0.000	0.015	0.155	0.017

a is χ2 value, b is F value, c is t value

After seven rounds of reading, the overall trend of diagnostic accuracy gradually improved in all three groups, with the largest increase in Group 2. Except for the third round, there were significant differences in the diagnostic compliance rates among the three groups. There was no statistical difference between Groups 2 and 3 at rounds 3, 4, 6, and 7. Table 3 presents the relevant test values.

**Table 3:** Comparison of diagnostic compliance rate among three groups of observers (%)

Group		Diagnostic compliance rate(%)						
		1	2	3	4	5	6	7
1		68.09	70.37	73.89	72.64	79.57	74.71	80.28
2		67.73	73.15	78.03	78.15	80.39	82.40	82.98
3		74.00	78.19	77.07	80.74	85.77	85.08	85.80
entirety	*χ* ^*2*^	9.966	15.836	4.900	17.292	15.075	31.981	9.198
	*P*	0.007	0.000	0.086	0.000	0.001	0.000	0.010
1vs2	*χ* ^*2*^	0.024	1.850	4.420	7.287	0.203	15.068	2.070
	*P*	0.876	0.174	0.036	0.007	0.652	0.000	0.150
2vs3	*χ* ^*2*^	8.041	6.154	0.028	1.824	9.751	2.264	2.566
	*P*	0.005	0.013	0.868	0.177	0.002	0.132	0.109
1vs3	*χ* ^*2*^	7.182	14.706	3.750	16.328	12.740	28.737	9.205
	*P*	0.007	0.000	0.053	0.000	0.000	0.000	0.003

### Kappa consistency test results for all the trainees

**Fig. 3:**
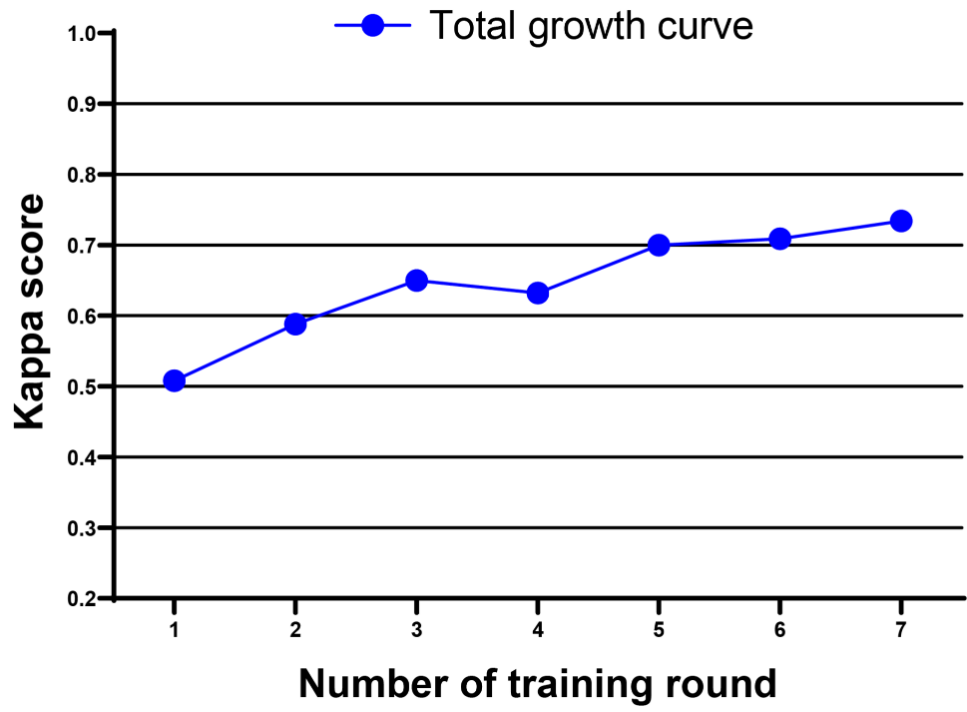
Growth curve of the average kappa scores for the three groups of training participants. The abscissa is the number of training rounds, and the ordinate is the Kappa score

Table 4 presents the average harmonic means and mean kappa scores in the test. After seven rounds of training, the average kappa score increased from 0.508 to 0.734. The mean kappa scores of the first four rounds were 0.595, implying medium consistency. The mean kappa score of the last three rounds increased to 0.714, signifying significant consistency. An escalating trend in diagnostic consistency was observed. Figure 3 illustrates the growth curve for training.

**Table 4:** Average harmonic mean and kappa scores of each round

	Number of rounds						
	1	2	3	4	5	6	7
Mean value of kappa scores	0.508	0.588	0.650	0.632	0.700	0.709	0.734
Average harmonic mean	0.436	0.562	0.661	0.619	0.669	0.701	0.732

### Kappa consistency test results for each group

**Fig. 4:**
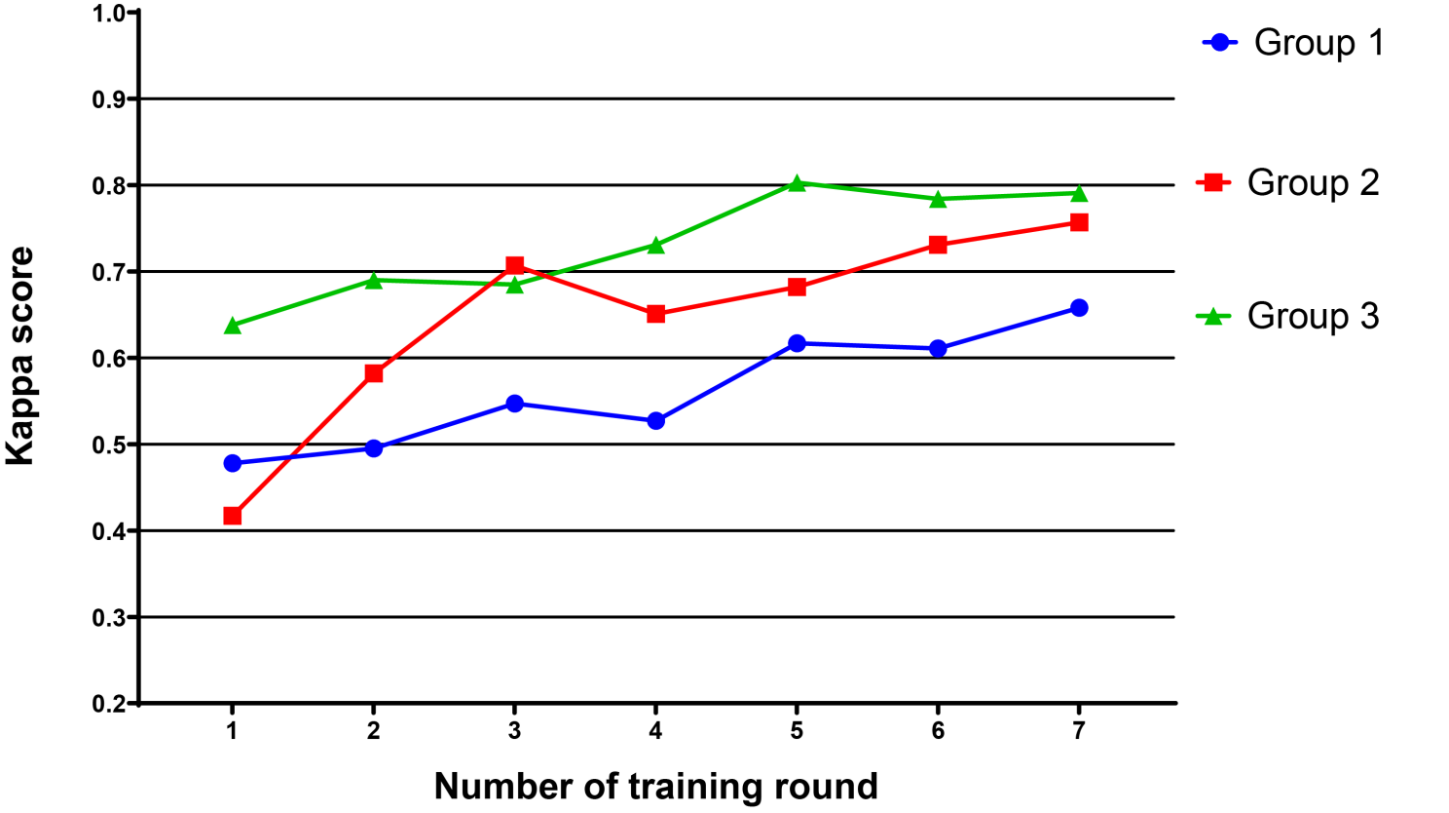
Growth curve of the average kappa scores for each of the three groups. The abscissa is the number of training rounds, and the ordinate is the Kappa score

A total of 18 participants were divided into three groups. The mean kappa score of each group was calculated separately. After seven rounds of training, the average kappa score of Groups 1, 2, and 3 increased from 0.478 to 0.658, 0.417 to 0.757, 0.638 to 0.791, respectively (Table 5). Figure 4 illustrates the curves according to the kappa scores of the three groups.

**Table 5:** Mean kappa scores of the three groups

Group	Number of rounds						
	1	2	3	4	5	6	7
1	0.478	0.495	0.547	0.527	0.617	0.611	0.658
2	0.417	0.582	0.707	0.651	0.682	0.731	0.757
3	0.638	0.690	0.685	0.731	0.803	0.784	0.791

## Discussion

Medical imaging— a highly pratical discipline— is akin a bridge connecting basic medicine and clinical medicine [14]. In practice, the detection of pulmonary nodules requires a certain level of clinical experience, which is immensely significant for the risk assessment of lung cancer. The increasing rate of chest CT screening and the concomitant heavy reading work exacerbates the fatigue of radiologists. Consequently, the risk of missed diagnosis or misdiagnosis increases [15]. The emergence of AI has significantly improved the diagnostic accuracy of radiologists. AI has greater than 95% diagnostic sensitivity and specificity [16], which is a pulmonary nodule detection model built on the architecture of the convolutional neural network [17].

Previous studies have mainly discussed the principle and algorithm steps of AI [18, 19]; very few studies have explored how to reasonably apply AI in clinical medical imaging work and how to integrate it with clinical teaching. Many revolutionary education models have been developed to adress the mismatch between the growing personalised requirements of students and the scarce faculty. Among them, the ‘AI + education’ model has considerably addressed this issue. According to Fleichner’s update guidelines for pulmonary nodules [20], the number, diameter, density and shape of lung nodules are significant indicators for the follow-up of nodules. Therefore, this study mainly evaluated the teaching effect of radiology interns’ application of AI training in the detection and diagnosis of pulmonary nodules from the perspectives of their location, number and benign and malignant properties.

Our subjects were divided into three groups. The Groups 1 and 2, included medical imaging students who had acquired the basic knowledge before the reading training and had not participated in clinical work. Group 3 comprised junior radiology residents with some basic diagnostic experience of radiology. The initial kappa scores of Groups 1 and 2, were similar, and lower than that of Group 3, indicating the differences in the knowledge and experience base of readers. The kappa score did not increase linearly each time, possibly because the difficulty level was not exactly consistent with each loaded images, causing a bias in the results. The mean kappa score of the three groups increased from 0.595 for the first four rounds to 0.714 for the last three rounds, indicating medium consistency to a significant improvement in the overall reading consistency after training. The diagnostic compliance rate of the three groups also increased simultaneously, with more significant increases in Group 2, indicating a significant improvement in the overall reading accuracy after AI training.

As the training progressed, the diagnostic consistency gap between Groups 2 and 3 narrowed. Thus, after the AI-reading training, even medical imaging students without clinical experience in radiology department can acquire familiarity with Lung-RADS reading rules and achieve a certain diagnostic accuracy. Similarly, after seven rounds of reading training, the kappa scores of Group 1, which did not have the AI software for auxiliary diagnosis, also improved to varying degrees. However, the overall trend and the kappa values were lower than those of Groups 2 and 3.

With the AI-assisted training, both junior radiology residents and medical imaging students improved the detection sensitivity of different pulmonary nodules. The possible reasons are as follows: (1) The considerable sensitivity of AI [21] could indicate the detailed information of the lesion in the software, and enhance the understanding of the knowledge point. (2) We believe that the junior radiology residents and medical imaging students lack the ability to distinguish the sub-solid nodules from other infectious foci, vascular sections, scar foci and hypostatic effects. AI training and repeated comparison cound enable the students to rapidly learn and summarise knowledge points in this area, improving the detection sensitivity. However, currently, AI cannot conduct subjective screening, and therefore, it will attribute some lung markings, low-density vascular sections, and even some lymph nodes with smaller diameters as nodules [22–24]. Thus, during lectures and discussions, teacher should remind students to remain attentive.

Additionally, our study identified that compared to other density pulmonary nodules, the three groups of trainees were more likely to miss the diagnosis of pGGN. Pathologically, persistent lung pGGN is mostly lung adenocarcinoma or its precancerous lesions [25]. Studies have shown that the probability of pGGN becoming lung adenocarcinoma is higher than that of solid nodules [26, 27]. Therefore, AI has immense clinical significance as it could help improve imaging physicians’ pGGN detection efficiency. To sum up, compared to the traditional medical imaging training methods, the radiologists employed professional knowledge and clinical experience—both powered with AI—to effectively screen and filter out those false positive nodules, this has immense clinical benefits.

Moreover, the detection sensitivity of junior radiology residents towards solid nodules and nodules with diameter less than 5 mm is higher than that of medical imaging students. Also, the number of false positive nodules is significantly less than that of the latter. This indicates that junior radiology residents have mastered certain basic imaging knowledge and film-reading skills and can more accurately identify solid pulmonary nodules and pulmonary nodules with diameter less than 5 mm, thus reducing the number of false positive nodules. However, owing to insufficient work experience, training, and clinical thinking, their detection sensitivity towards solid nodules and pulmonary nodules with diameter less than 5 mm is not high.

This study has a few limitations. First, the AI system cannot provide correct scoring answer, and retrospective learning of images should be uniformly performed after each label. This will improve the reading learning efficiency. Second, as the sample size is small, the measurement data and statistical results may be biased. Additionally, the results of this study reflect the performance of AI software with specific parameters and specific algorithms; they cannot verify the results of other AI software. Extensive and multi-centre joint studies are required in the future.

## Conclusions

In conclusion, for junior radiology residents and medical imaging students, AI-assisted software can improve the detection efficiency regarding different pulmonary nodules in CT. AI tools can serve as an aide in medical education alongside the classical theoretical and practical medical education. AI-based teaching may create issues regarding the false negative/false positive results. It could also raise ethical concerns.

## Data Availability

The data and materials used and analyzed during the study are available from the corresponding author on reasonable request.

## Abbreviations

AI: Artificial intelligence
PACS: Picture archiving and communication system
CT: Computed tomography
Lung-RADS: Lung CT Screening Reporting and Data System
(pGGN): Pure ground glass nodule
(mGGN): Mixed ground glass nodule
